# Supplementary material for: Investigating sustainable development in transportation enterprises: Novel insights from new institutional economics and human capital theory. Evidence from HCM, Vietnam
Source: PLoS One. 2025 Nov 17;20(11):e0333393. doi: 10.1371/journal.pone.0333393 (PMC12622828; doi:10.1371/journal.pone.0333393)
Supplement: S1 Appendix — (DOCX) [file pone.0333393.s001.docx]

**S1_Appendix. Demographic and professional characteristics of pilot survey respondents**

| **Criterion** | **Sample size** | **N** | **Percentage** |
| --- | --- | --- | --- |
| **Gender** | 50 |  |  |
| Male |  | 35 | 70% |
| Female |  | 15 | 30% |
| **Education** | 50 |  |  |
| College |  | 10 | 20% |
| University |  | 38 | 76% |
| Postgraduate |  | 2 | 4% |
| **Age** | 50 |  |  |
| 26-35 years old |  | 10 | 20% |
| 36-45 years old |  | 28 | 56% |
| 46-55 years old |  | 9 | 18% |
| Over 55 years old |  | 3 | 6% |
| **Position** | 50 |  |  |
| General manager |  | 20 | 40% |
| Manager |  | 30 | 60% |
| **Working Experience** | 50 |  |  |
| 5-10 years |  | 7 | 14% |
| 11-15 years |  | 18 | 36% |
| 16-20 years |  | 15 | 30% |
| Over 20 years |  | 10 | 20% |
| **Type of Business** | 50 |  |  |
| Limited liability company |  | 25 | 50% |
| Joint stock company |  | 13 | 26% |
| Private company |  | 5 | 10% |
| Company with foreign investment capital |  | 7 | 14% |
| **Labour Size** | 50 |  |  |
| Under 11 employees |  | 35 | 70% |
| 11-50 employees |  | 8 | 16% |
| 51-100 employees |  | 4 | 8% |
| 101-200 employees |  | 2 | 4% |
| Over 200 employees |  | 1 | 2% |
| **Operating Time** | 50 |  |  |
| Less than 1 year |  | 2 | 4% |
| 1-5 years |  | 11 | 22% |
| 6-10 years |  | 14 | 28% |
| 11-15 years |  | 18 | 36% |
| More than 15 years |  | 5 | 10% |
