# Supplementary material for: Investigating sustainable development in transportation enterprises: Novel insights from new institutional economics and human capital theory. Evidence from HCM, Vietnam
Source: PLoS One. 2025 Nov 17;20(11):e0333393. doi: 10.1371/journal.pone.0333393 (PMC12622828; doi:10.1371/journal.pone.0333393)
Supplement: S2 Appendix — (DOCX) [file pone.0333393.s002.docx]

**S2_Appendix. Cronbach's alpha test results of pilot test**

| **Item** | **Item-rest correlation (1st)** | **Alpha (1st)** | **Item-rest correlation (2rd)** | **Alpha (2rd)** |
| --- | --- | --- | --- | --- |
| Challenging institutions – component 1 | 0.838 | 0.903 |  |  |
| Challenging institutions – component 2 | 0.664 | 0.925 |  |  |
| Challenging institutions – component 3 | 0.819 | 0.906 |  |  |
| Challenging institutions – component 4 | 0.753 | 0.915 |  |  |
| Challenging institutions – component 5 | 0.784 | 0.911 |  |  |
| Challenging institutions – component 6 | 0.838 | 0.903 |  |  |
| Scale reliability coefficient of challenging institutions: 0.925 |  |  |  |  |
| Supporting institutions – component 1 | 0.549 | 0.818 | 0.514 | 0.919 |
| Supporting institutions – component 2 | 0.775 | 0.769 | 0.791 | 0.855 |
| Supporting institutions – component 3 | 0.694 | 0.789 | 0.760 | 0.863 |
| Supporting institutions – component 4 | 0.783 | 0.775 | 0.812 | 0.854 |
| Supporting institutions – component 5 | 0.779 | 0.770 | 0.841 | 0.844 |
| Supporting institutions – component 6 | 0.173 | 0.892 | **Scale reliability coefficient of positive institutions: 0.892** | |
| Scale reliability coefficient of supporting institutions: 0.833 |  |  |  |  |
| Human capital - component 1 | 0.805 | 0.833 |  |  |
| Human capital - component 2 | 0.818 | 0.829 |  |  |
| Human capital - component 3 | 0.491 | 0.894 |  |  |
| Human capital - component 4 | 0.758 | 0.842 |  |  |
| Human capital - component 5 | 0.857 | 0.824 |  |  |
| Human capital - component 6 | 0.430 | 0.891 |  |  |
| Scale reliability coefficient of human capital: 0.875 |  |  |  |  |
| Social development - component 1 | 0.610 | 0.875 | 0.626 | 0.896 |
| Social development - component 2 | 0.806 | 0.856 | 0.834 | 0.876 |
| Social development - component 3 | 0.676 | 0.868 | 0.647 | 0.894 |
| Social development - component 4 | 0.841 | 0.853 | 0.854 | 0.874 |
| Social development - component 5 | 0.852 | 0.853 | 0.882 | 0.872 |
| Social development - component 6 | 0.737 | 0.864 | 0.750 | 0.885 |
| Social development - component 7 | 0.615 | 0.874 | 0.633 | 0.895 |
| Social development - component 8 | 0.358 | 0.894 | 0.330 | 0.920 |
| Social development - component 9 | 0.257 | 0.902 | **Scale reliability coefficient of social development: 0.902** | |
| Scale reliability coefficient of social development: 0.885 |  |  |  |  |
| Economic development - component 1 | 0.901 | 0.953 |  |  |
| Economic development - component 2 | 0.835 | 0.956 |  |  |
| Economic development - component 3 | 0.923 | 0.952 |  |  |
| Economic development - component 4 | 0.746 | 0.960 |  |  |
| Economic development - component 5 | 0.911 | 0.953 |  |  |
| Economic development - component 6 | 0.814 | 0.957 |  |  |
| Economic development - component 7 | 0.796 | 0.958 |  |  |
| Economic development - component 8 | 0.814 | 0.957 |  |  |
| Economic development - component 9 | 0.796 | 0.958 |  |  |
| Scale reliability coefficient of economic development: 0.961 |  |  |  |  |
| Environmental development - component 1 | 0.852 | 0.935 | 0.855 | 0.951 |
| Environmental development - component 2 | 0.938 | 0.931 | 0.948 | 0.946 |
| Environmental development - component 3 | 0.938 | 0.931 | 0.948 | 0.946 |
| Environmental development - component 4 | 0.873 | 0.934 | 0.885 | 0.949 |
| Environmental development - component 5 | 0.908 | 0.932 | 0.919 | 0.948 |
| Environmental development - component 6 | 0.904 | 0.932 | 0.909 | 0.948 |
| Environmental development - component 7 | 0.896 | 0.933 | 0.901 | 0.948 |
| Environmental development - component 8 | 0.529 | 0.948 | 0.512 | 0.965 |
| Environmental development - component 9 | 0.231 | 0.957 |  |  |
| Environmental development - component 10 | 0.529 | 0.948 | 0.572 | 0.969 |
| Scale reliability coefficient of environmental development: 0.945 |  |  | **Scale reliability coefficient of environmental development: 0.958** | |
