# Supplementary material for: Investigating sustainable development in transportation enterprises: Novel insights from new institutional economics and human capital theory. Evidence from HCM, Vietnam
Source: PLoS One. 2025 Nov 17;20(11):e0333393. doi: 10.1371/journal.pone.0333393 (PMC12622828; doi:10.1371/journal.pone.0333393)
Supplement: S3 Appendix — (DOCX) [file pone.0333393.s003.docx]

**S3_Appendix. Demographic and professional characteristics of offical survey respondents**

| **Criterion** | **Sample size** | **N** | **Percentage** |
| --- | --- | --- | --- |
| **Gender** | 354 |  |  |
| Male |  | 210 | 59% |
| Female |  | 144 | 41% |
| **Education** | 354 |  |  |
| College |  | 90 | 25% |
| University |  | 220 | 62% |
| Postgraduate |  | 44 | 12% |
| **Age** | 354 |  |  |
| 26-35 years old |  | 100 | 28% |
| 36-45 years old |  | 150 | 42% |
| 46-55 years old |  | 70 | 20% |
| Over 55 years old |  | 34 | 10% |
| **Position** | 354 |  |  |
| General manager |  | 140 | 40% |
| Manager |  | 214 | 60% |
| **Working Experience** | 354 |  |  |
| 5-10 years |  | 50 | 14% |
| 11-15 years |  | 120 | 34% |
| 16-20 years |  | 100 | 28% |
| Over 20 years |  | 84 | 24% |
| **Type of Business** | 354 |  |  |
| Limited liability company |  | 150 | 42% |
| Joint stock company |  | 100 | 28% |
| Private company |  | 54 | 15% |
| Company with foreign investment capital |  | 50 | 14% |
| **Labour Size** | 354 |  |  |
| Under 11 employees |  | 243 | 69% |
| 11-50 employees |  | 90 | 25% |
| 51-100 employees |  | 14 | 4% |
| 101-200 employees |  | 5 | 1% |
| Over 200 employees |  | 2 | 1% |
| **Operating Time** | 354 |  |  |
| Less than 1 year |  | 20 | 6% |
| 1-5 years |  | 80 | 23% |
| 6-10 years |  | 100 | 28% |
| 11-15 years |  | 120 | 34% |
| More than 15 years |  | 34 | 10% |
