# Supplementary material for: Investigating sustainable development in transportation enterprises: Novel insights from new institutional economics and human capital theory. Evidence from HCM, Vietnam
Source: PLoS One. 2025 Nov 17;20(11):e0333393. doi: 10.1371/journal.pone.0333393 (PMC12622828; doi:10.1371/journal.pone.0333393)
Supplement: Attachment — Submitted filename: PONE-D-25-32321 Review comments.pdf [file pone.0333393.s007.pdf]

Specific comments for PLOS ONE Manuscript Number PONE-D-25-32321

Manuscript Number: PONE-D-25-32321

Full Title: Investigating how institutions and human capital influence sustainable transportation development, and how it responds in return: evidence from an urban transport hub in an emerging economy

Decision: Major revision

1). Summary of the manuscript:

The study examines the ways in which human capital and institutional factors impact the development of sustainable transportation in Ho Chi Minh City, Vietnam, and how these sustainability factors in turn affect the same drivers. The study tests several hypotheses within a conceptual model based on Becker's human capital theory and North's institutional theory using PLS-SEM on data from 354 transportation enterprises. The study reveals a web of reciprocal connections between the environmental, economic, and social facets of sustainable transportation, human capital traits, and institutional quality.

2). General assessment

Originality: In the literature on sustainable transportation, the idea of bidirectional influence is fresh and well-developed. It broadens the use of theories of human capital and institutions to include sustainability and transportation.

Scientific rigor: Strong theoretical foundation and a sound empirical approach utilizing PLS-SEM are hallmarks of scientific rigor. However, some theoretical framing and methodological details require clarification.

Clarity: The text is sometimes too wordy and dense. It would be possible to streamline several sections of the text without sacrificing content.

Fit for PLOS ONE: Fulfills the journal's emphasis on scientific rigor rather than novelty in and of itself, although it needs to be revised for coherence, clarity, and interpretative accuracy.

3). Major concerns

1. Using PLS-SEM excessively without adequate model justification
   The manuscript occasionally suggests confirmatory analysis, even though PLS-SEM is suitable for exploratory theory-building. Whether this study is exploratory or confirmatory needs to be made clear by the authors.

For complete transparency, include measurement model tables (loadings, AVE, CR, and HTMT) in the main body of the text or as supplementary materials.

2. Although the theoretical framework is robust, it lacks integration. Becker's human capital theory and North's institutional theory are both presented effectively on their own, but they are not well integrated or synthesized, which is the most important contribution of this study.
   Add a synthesis paragraph outlining the potential interactions between human capital and institutions that could impact sustainability.

3. Definitions and operationalizations of constructs that are too general. The dichotomy of "positive" and "negative" institutional factors is overly simplistic. The subtleties of institutional economics are oversimplified by this binary classification. Think about creating multidimensional subscales (such as transparency, corruption, and enforcement) or utilizing institutional quality indices.
   Items like "innovation" and "creativity," which are more closely related to firm-level capabilities than to human capital in and of themselves, are included in the human capital construct. Give a clearer explanation for their inclusion.

4. Ambiguity in causal inference: Despite strong statistical modeling, causality is not established. Although the authors refer to "bidirectional influence," they treat correlations as effects
   Make it clear that unless there are compelling assumptions (such as experimental designs or longitudinal data), these are associations rather than causality.

5. Nuance is required in the interpretation of results. Effect sizes and their practical significance are not discussed in the interpretation of β coefficients (such as 0.328 or -0.154).
   Include a section discussing how effect sizes - rather than just statistical significance – influence policy implications.

4). Minor concerns

1. Language: The manuscript needs to be English edited because it contains awkward wording, redundant information, and excessively formal constructions. Suggest expert language polishing.
2. Length: Close to 20,000 words, the paper is incredibly lengthy. Suggest condensing the literature review and discussion without sacrificing content.
3. Figures and Tables: A number of the tables, such as the measurement model and survey scales, are excessively detailed and ought to be relegated to the supplemental material.
4. References: Verify that all citations (such as [63], [64], and [7]) are accurate and formatted in accordance with PLOS ONE guidelines.

5). Specific comments

1. *Abstract:* Well-organized, although it should be made clear that "bidirectional relationships" are statistical associations rather than causative ones.

2. *Introduction:* Strong context-setting in the introduction; however, to prevent reader fatigue, it could be condensed by 20% to 30%.
3. *Literature review:* Needs tighter synthesis, but impressive depth. Don't use several paragraphs to reiterate what institutional theory is.
4. *Methods:* Provide a more thorough justification for sample representativeness. Is 354 of 3,908 sufficient for generalization? Explain why two stages of data collection were required as well.
5. *Results:* Tables are understandable, but there is no explanation of effect size.
6. *Discussion:* The introduction and this section are similar. Highlight the most important discoveries and make a clear connection between them and your research questions and hypotheses.

Explanations for Review Questions

1. Is the manuscript technically sound, and do the data support the conclusions?

   Assessment: Partially - revisions needed

   Although the design and execution of the manuscript are technically sound, not all of the conclusions are entirely supported by the data as they stand at this time. Some theoretical concepts require more precise definitions, and statements regarding bidirectional influence need to be rephrased to account for cross-sectional SEM's limitations. The study might make a significant empirical contribution with these changes.

   Strengths (technically sound components):

   Methodology: Robust quantitative methodology is used. Using Partial Least Squares given the complexity of the proposed relationships and the type of latent constructs involved, structural equation modeling, or PLS-SEM, is a suitable method.

   Adequate Sample Size: To ensure statistical power, the final sample of 354 transportation enterprises is larger than the typically recommended minimum sample size for PLS-SEM (200–300).

   Measurement Model Validation: Although the manuscript contains reliability and validity checks (such as Cronbach's alpha, AVE, and HTMT), some specific values are disorganized and buried deep within the text.

   Bidirectional Hypotheses: The model adds theoretical value and is conceptually ambitious by testing both forward and reverse relationships.

Concerns/limitations

Causal Inference: Although the study refers to bidirectional "influences," it uses cross-sectional data. This wording suggests causation, which the data neither statistically nor chronologically support. To take associational findings into account, authors must rephrase the manuscript.

Ambiguities in Construct Validity: It is conceptually flawed to categorize institutional factors as "positive" or "negative." This dichotomy oversimplifies the effects of institutions, which frequently behave in complex, context-dependent ways.

The theoretical consistency is weakened by the fact that some human capital metrics (such as "creativity") overlap with organizational innovation rather than individual capability.

Interpretation of Effect Size: The conclusions lack adequate discussion of the magnitude or practical relevance of the effects (e.g., $\beta = 0.12$ vs. $\beta = 0.60$) and mainly rely on statistical significance (p-values).

Weaker R^2 values: for some dependent constructs (e.g., economic development $R^2 = 0.061$) suggest limited explanatory power, yet the paper draws strong policy conclusions in these areas.

2. Has the statistical analysis been performed appropriately and rigorously?

Assessment: Yes (largely), but needs some improvements

Given the following, PLS-SEM is a suitable choice:

- The exploratory nature of the model
- Multiple latent constructs
- Non-normal assumptions for data
- N = 354 is a moderate sample size.

In terms of the measurement model, authors report Cronbach's alpha, CR, AVE, and HTMT ratios to assess internal consistency, convergent, and discriminant validity. These exceed conventional thresholds ($\alpha > 0.70$, AVE > 0.50, HTMT < 0.85), indicating sound measurement properties. VIF values were examined to test multicollinearity, and problematic indicators have been removed.

In terms of the structural model assessment, authors evaluate path coefficients, p-values, R^2 values, and Q^2_predict, which reflect appropriate rigor. The model shows moderate explanatory power for key endogenous variables (e.g., $R^2 = 0.458$ for environmental development, 0.459 for human capital).

However, following concerns need to be addressed.

a.) Sample size vs. model complexity:
In terms of model stability in PLS-SEM, the model's 50+ indicators and 6 latent

constructs may be close to the maximum amount that a sample of 354 can manage. The authors should report and discuss power or model parsimony and provide a clearer explanation of the model complexity.

b.) Limitations of cross-sectional data:
The bidirectional paths are tested simultaneously using cross-sectional data, which introduces the risk of reciprocal causality bias. The authors should not imply directional or temporal causality, even if the model shows good fit. This needs a clear disclaimer in the limitations section.

c.) PLS-Predict and Predictive Power:
Although PLS-Predict is mentioned, the reporting lacks sufficient depth (e.g., benchmark model RMSE, Q^2 values for each indicator). Authors should provide full PLS-Predict outputs as Supplementary Information to verify out-of-sample predictive relevance.

d.) Lack of Mediation or Moderation Analysis:
Given the conceptual richness of the framework (e.g., institutions and human capital interacting), the lack of mediation/moderation testing may limit insight. While not essential, the authors might discuss whether they tested or considered such effects.

3. Have the authors made all data underlying the findings in their manuscript fully available?

Assessment: No

Authors have only provided survey design, sampling strategy, and data collection process together with descriptives of the data. There is no reference in the text to a URL or DOI or a supplementary dataset. However, they have provided the data from the pilot survey as "supporting information" which is not adequate to verify the PLS-SEM results.

In the revised version, authors should include a data availability statement with a link to repository or DOI.

4. Is the manuscript presented in an intelligible fashion and written in standard English?

Assessment: No

The manuscript is intelligible but needs significant language and structural improvements. Clarity would be enhanced by shortening long sentences, eliminating redundancy, moving technical appendices to supplementary files, and using more concise academic phrasing. A professional copy-edit or substantial language review is recommended before publication.

The manuscript suffers from verbosity and redundancy. Sentences are often too long, with excessive qualifiers and formal constructions. Some sentences are grammatically

correct but stylistically awkward, especially with overuse of passive voice and prepositional stacking. The word count should be too much for a PLOS ONE manuscript.

Suggested improvements:

- Literature review and methodology sections could be condensed by 25-30% without losing substance.
- Several paragraphs repeat concepts that are already introduced.
- Some tables could be removed and made available as supplementary information

Language

The manuscript is written in standard English but not polished academic English. It would benefit from professional language editing, especially to improve clarity, conciseness, and tone.
