# Supplementary material for: Investigating sustainable development in transportation enterprises: Novel insights from new institutional economics and human capital theory. Evidence from HCM, Vietnam
Source: PLoS One. 2025 Nov 17;20(11):e0333393. doi: 10.1371/journal.pone.0333393 (PMC12622828; doi:10.1371/journal.pone.0333393)
Supplement: Attachment — Submitted filename: Review.docx [file pone.0333393.s006.docx]

Review of the Research Article Titled: **Investigating How Institutions and Human Capital Influence Sustainable Transportation Development, and How It Responds in Return: Evidence from an Urban Transport Hub in an Emerging Economy**

In this study the author has assessed the Ho Chi Minh City transportation system in the background of environmental sustainability. The researcher has tried to look into the practical issue of the city transportation from the perspectives of the institutions and public response. S/He actually helps to develop a practicable model that has been already in place in other advance parts of the world. North’s institution theory aptly applied to tell that how the sense of transportation is helping the citizens behavior. The three dimensions; economic, social and environmental are integrated to work closely. Human capital theory is applied in the writing that how people can better respond to the Ho Chi Minh City transportation as an institution.

The author works seems original and very much according to the standards of research but with two shortcomings;

1. The author has divided the study according to the two theories. This creates an issue to naturally combine the theories in the end. Better if the author has discussed both of the theories in a debate form to draw a single list of hypotheses and research gaps,
2. The study is too long with a long list of references as well. It could have been squeezed.

Final Note. Despite its shortcomings, the research is very much original and interesting. I would hereby recommend it to be published.

Dr. Khurshaid,

Department of International Relations

University of Peshawar

Pakistan

[Khurshid4ir@uop.edu.pk](mailto:Khurshid4ir@uop.edu.pk)
