# Supplementary material for: Investigating sustainable development in transportation enterprises: Novel insights from new institutional economics and human capital theory. Evidence from HCM, Vietnam
Source: PLoS One. 2025 Nov 17;20(11):e0333393. doi: 10.1371/journal.pone.0333393 (PMC12622828; doi:10.1371/journal.pone.0333393)
Supplement: Attachment — Submitted filename: 4. Response to Reviewers 17 AUG 25.docx [file pone.0333393.s010.docx]

Vu Thi Kim Hanh (Corresponding author)
Email: [hanhvtk20702@sdh.uel.edu.vn](mailto:hanhvtk20702@sdh.uel.edu.vn)
Nguyen Hong Nga (Co - corresponding author)
Email: [nganh@uel.edu.vn](mailto:nganh@uel.edu.vn)
1. University of Economics and Law, Ho Chi Minh City, Vietnam.
2. Vietnam National University, Ho Chi Minh City, Vietnam.

17 August 2025

**Editor-in-Chief**
PLOS ONE

Dr Wong Ming Wong, Academic Editor

**RESPONSE TO REVIEWER LETTER**

Manuscript Number: PONE-D-25-32321

Previous title: Investigating How Institutions and Human Capital Influence Sustainable Transportation Development, and How It Responds in Return: Evidence from an Urban Transport Hub in an Emerging Economy

Revised title: Investigating Sustainable Development in Transportation Enterprises: Novel Insights from New Institutional Economics and Human Capital Theory. Evidence from HCM, Vietnam

Dear Dr Wong Ming Wong, Academic Editor, and Esteemed Reviewers,

We sincerely appreciate the opportunity to revise and resubmit our manuscript. We are grateful for the constructive feedback provided by you and the reviewers, which has guided us in strengthening the clarity, coherence, and overall quality of the manuscript. Your detailed insights have been invaluable in helping us refine both the conceptual framework and empirical presentation of our study.

As part of this resubmission, we would like to note a revision to the manuscript title. The title has been changed to “Investigating Sustainable Development in Transportation Enterprises: Novel Insights from New Institutional Economics and Human Capital Theory. Evidence from HCM, Vietnam***”*** to ensure alignment with our officially registered research funding documentation. This adjustment does not affect the theoretical framework, methodology, or findings of the study, which remain fully consistent with the previously reviewed version. The revised title aims to maintain clarity while meeting institutional and funding requirements.

Below, we provide a detailed, point-by-point response to each reviewer comment. We address Reviewer 1's comments first, in the order they were presented, followed by responses to Reviewer 2’s comments. All revisions have been incorporated into the updated manuscript, with changes clearly marked using track changes for your review.

### ****REVIEWER 1:****

### Reviewer 1’s Comment 1:

### The author has divided the study according to the two theories. This creates an issue to naturally combine the theories in the end. Better if the author has discussed both of the theories in a debate form to draw a single list of hypotheses and research gaps,

### ****Author’s Response to Reviewer 1 – Comment 1:****

Thank you very much for this insightful and constructive suggestion. We fully agree that integrating the two theoretical perspectives more cohesively strengthens the conceptual coherence of the study. In response, we have revised the manuscript to present **institutional theory** and **human capital theory** in an integrated, debate-oriented form that highlights their interdependencies rather than treating them as separate analytical silos.

Specifically, we have a revised subsection titled ***“2.2. Integrating Institutional and Human Capital Theories: A Dual-Lens Approach to Sustainable Transportation Development”*** (see revised manuscript, pages 5 - 11; highlighted in blue and green). This section engages both theories in dialogue, emphasizing how institutional structures are associated with the sustainable transportation development, and also integrated with capital, and vice versa.

For instance, while North’s institutional theory addresses the formal and informal rules governing behaviour, Becker’s human capital theory explains how individuals interpret and respond to these institutional contexts. We elaborate on how enabling institutions (e.g., regulatory incentives, digital infrastructure) promote the development and retention of human capital, and how skilled personnel, in turn, reinforce or challenge institutional norms through innovation, leadership, and knowledge sharing. Conversely, constraining institutions (e.g., corruption, rigid bureaucracy) can limit the impact of even highly capable individuals. In return, empowered human capital can act as a catalyst for institutional improvement.

This conceptual integration directly informed our development of a unified **research gap discussion, main contributions**, and a **consolidated set of hypotheses** (see revised manuscript, pages 9 - 11). The hypotheses reflect the bidirectional associations between institutional factors, human capital, and the three dimensions of sustainable transportation development, economic, social, and environmental. We have structured them to capture both direct and reciprocal relationships, as recommended.

We sincerely hope this revised and integrated approach addresses your concern and enhances the theoretical coherence and contribution of the manuscript. Thank you again for your valuable guidance.

**Reviewer 1’s Comment 2:**

The study is too long with a long list of references as well. It could have been squeezed.

**Author’s Response to Reviewer 1 – Comment 2:**

We sincerely thank you for your valuable observation regarding the excessive length of the manuscript and the extensive reference list. In response, and in alignment with Reviewer 2’s related suggestions, we undertook a thorough and careful revision of the entire manuscript to improve conciseness and focus.

The revised manuscript now contains **13,208 words** in total: **10,176 words** in the main text (including author information) and **3,032 words** in the references. For comparison, the previous version had **17,612 words** in total, comprising **13,896 words** (including Tables and author information) in the main text and **3,716 words** in the references.

Reviewer 2 recommended that some of the tables, which were considered excessively detailed, be moved to supplemental material. After reviewing all nine tables, we decided to relocate **all** of them to the single Supporting Material file. This decision was made for the sake of consistency and to further enhance readability, ensuring that readers can focus more easily on the main findings and discussions.

Additionally, to maintain academic rigor and credibility, we carefully reviewed the reference list and removed **34 references** that did not substantially contribute to the manuscript. This includes works from journals such as Sustainability, Atmosphere, and Energies (published by MDPI and listed on Beall’s List), as well as items not indexed in Scopus or Web of Science (*e.g., J Trust and MIS Q, Lit Verlag,* ITS & Transport Management Supplement). Sources of limited relevance were also excluded.

Instead, we supplemented the manuscript with several **new, high-quality references** to address specific points raised by Reviewer 2. These additional sources were carefully selected to strengthen the theoretical foundation, support the revised arguments, and ensure the scholarly robustness of the manuscript.

The list of removed references is provided below:

1. E. Macioszek, A. Granà, P. Fernandes, and M. C. Coelho, "New perspectives and challenges in traffic and transportation engineering supporting energy saving in smart cities—A multidisciplinary approach to a global problem," Energies, vol. 15, no. 12, Art. no. 4191, Jun. 2022. [Online]. Available: <https://doi.org/10.3390/en15124191>
2. Torosyan E, Amiryan S, Yesayan S. Development of human capital management system in the transportation industry. E3S Web Conf. 2020;164:10012. <https://doi.org/10.1051/e3sconf/202016410012>.
3. Ahuja S, Panigrahi BK, Dey N, Rajinikanth V, Gandhi TK. Deep transfer learning-based automated detection of COVID-19 from lung CT scan slices. Appl Intell 2021;51:571-85.
4. Franco S. The influence of the external and internal environments of multinational enterprises on the sustainability commitment of their subsidiaries: A cluster analysis. J Clean Prod 2021;297:126654. <https://doi.org/10.1016/j.jclepro.2021.126654>.
5. Schumacher AE, et al. Global age-sex-specific mortality, life expectancy, and population estimates in 204 countries and territories and 811 subnational locations, 1950–2021, and the impact of the COVID-19 pandemic: A comprehensive demographic analysis for the Global Burden of Disease Study 2021. The Lancet. 2024.
6. H. N. K. Vu et al., “Poor Air Quality and Its Association with Mortality in Ho Chi Minh City: Case Study,” Atmosphere, vol. 11, no. 7, p. 750, 2020, doi: <https://doi.org/10.3390/atmos11070750>*.*
7. H. T. Do, “Energy Demand Model Towards Sustainable On-Road Transportation in Ho Chi Minh City,” in Proceedings of the 8th International Conference on Sustainable Urban Development, V. T. Ha, H. N. Nguyen, and H.-J. Linke, Eds. Singapore: Springer Nature Singapore, 2024, pp. 303–320, <https://doi.org/10.1007/978-981-99-8003-1_17>*.*
8. Pojani D, Stead D. Sustainable urban transport in the developing world: Beyond megacities. Sustainability 2015;7(6):7784-805. <https://doi.org/10.3390/su7067784>.
9. Singh A, Gurtu A, Singh RK. Selection of sustainable transport system: a case study. Manag Environ Qual 2021;32(1):100-13.
10. Hietanen S. Mobility as a Service. The new transport model? ITS & Transport Management Supplement. 2014;2–4.
11. Kamargianni M, Matyas M, Li W, Schäfer A. A critical review of new mobility services for urban transport. Transp Res Procedia. 2016;14:3294–3303.
12. Shaheen SA, Chan ND, Gaynor T, & Camel T. Shared mobility: Definitions, industry developments, and early understanding. UC Berkeley: Transportation Sustainability Research Center; 2015.
13. Anderson JM, Nidhi K, Stanley KD, Sorensen P, Samaras C, Oluwatola OA. Autonomous Vehicle Technology: A Guide for Policymakers. Rand Corporation; 2016.
14. Goodall NJ. Machine ethics and automated vehicles. In: Meyer G, Beiker S, eds. Road Vehicle Automation. Springer International Publishing; 2014:93–102.
15. Bimbraw K. Autonomous cars: Past, present and future. Proc 12th Int Conf Inf Control Autom Robot (ICINCO). 2015;1(1):191–198.
16. Kucharcikova A, Miciak M. Human capital management in transport enterprises with the acceptance of sustainable development in the Slovak Republic. Sustainability 2018;10:2530. <https://doi.org/10.3390/su10072530>.
17. Kucharcikova A, Miciak M. Human capital management in transport enterprise. MATEC Web Conf 2017;134:1-6.
18. Willaby HW, Costa DSJ, Burns BD, MacCann C, Roberts RD. Testing complex models with small sample sizes: A historical overview and empirical demonstration of what partial least squares (PLS) can offer differential psychology. Pers Individ Differ. 2015;84:73–8.
19. Rindfleisch A, Malter AJ, Ganesan S, Moorman C. Cross-sectional versus longitudinal survey research: Concepts, findings, and guidelines. J Mark Res 2008;45(3):261–79.
20. Henseler J, Ringle CM, Sinkovics RR. The use of partial least squares path modeling in international marketing. In: Sinkovics RR, Ghauri PN, eds. New Challenges to International Marketing. Advances in International Marketing. Emerald Group Publishing Limited; 2009:277-19.
21. Hair JF, Ringle CM, Sarstedt M. PLS-SEM: Indeed a silver bullet. J Mark Theory Pract 2011;19:139-52.
22. G. Shmueli, S. Ray, J. M. Velasquez Estrada, and S. B. Chatla, “The elephant in the room: Predictive performance of PLS models,” Journal of Business Research, vol. 69, no. 10, pp. 4552–4564, Oct. 2016, doi: [10.1016/j.jbusres.2016.03.049](https://doi.org/10.1016/j.jbusres.2016.03.049).
23. J. F. Hair, G. T. M. Hult, C. M. Ringle, and M. Sarstedt, “The use of partial least squares structural equation modeling in strategic management research: A review of past practices and recommendations for future applications,” Long Range Planning, vol. 45, no. 5–6, pp. 320–340, 2012.
24. M. Sarstedt, C. M. Ringle, and J. F. Hair, “Beyond a tandem analysis of SEM and PROCESS: Use PLS-SEM for mediation analyses!,” *Int. J. Market Res.*, vol. 62, no. 3, pp. 288–299, 2020.
25. G. Cho and J. Y. Choi, “An empirical comparison of generalized structured component analysis and partial least squares path modeling under variance-based structural equation models,” *Behaviormetrika*, vol. 47, pp. 243–272, 2020.
26. J. F. Hair and M. Sarstedt, “Explanation plus prediction—The logical focus of project management research,” *Project Manag. J.*, vol. 52, no. 4, pp. 321–329, 2021*.*
27. J. F. Hair, J. J. Risher, M. Sarstedt, and C. M. Ringle, “When to use and how to report the results of PLS-SEM,” European Business Review, vol. 31, no. 1, pp. 2–24, 2019. doi: 10.1108/EBR-11-2018-0203.
28. Lang A, Murphy H. Business and sustainability. Cham: Springer; 2014. <https://doi.org/10.1007/978-3-319-07239-5>.
29. Orzeszyna K, Tabaszewski R. The legal aspects of activities taken by local authorities to promote sustainable development goals: Between global and regional regulations in Poland. Lex Localis 2021;19(1):1–20.
30. Ledeneva A. Informal networks in post-communist economies: A “topographic map.” In: Lahusen T, Solomon PH Jr., editors. What is Soviet Now? Identities, legacies, memories. Berlin: Lit Verlag; 2008. p. 101-34.
31. Möllering G. Trust, calculativeness, and relationships: A special issue 20 years after Williamson's warning. J Trust 2014;4(1):1–21.
32. G. Shmueli and O. R. Koppius, “Predictive analytics in information systems research,” *MIS Q.*, vol. 35, no. 3, pp. 553–572, 2011.
33. E. E. Rigdon, “Rethinking partial least squares path modeling: In praise of simple methods,” Long Range Plann., vol. 45, no. 5–6, pp. 341–358, 2012.
34. J. F. Hair Jr., G. T. M. Hult, C. M. Ringle, M. Sarstedt, N. P. Danks, and S. Ray, "An Introduction to Structural Equation Modeling," in Partial Least Squares Structural Equation Modeling (PLS-SEM) Using R, Cham, Switzerland: Springer, 2021, pp. 1–29, doi: 10.1007/978-3-030-80519-7_1

**REVIEWER 2: 3) Major concerns**

**Reviewer 2:** 1. Using PLS-SEM excessively without adequate model justification. The manuscript occasionally suggests confirmatory analysis, even though PLS-SEM is suitable for exploratory theory-building. Whether this study is exploratory or confirmatory needs to be made clear by the authors.

**Authors response:**

Thank you for this important observation. We have clarified the methodology section in 3.6. Method Selection: Justification for PLS-SEM (Theoretical and methodological alignment), on **page 17** (highlighted in yellow). Specifically, we now explain that the study adopts a primarily exploratory approach grounded in theory-building, while also incorporating confirmatory elements through hypothesis testing. This justifies the use of PLS-SEM as an exploratory-confirmatory hybrid approach, supported by Hair et al. (2022)/[70].

**Reviewer 2:** For complete transparency, include measurement model tables (loadings, AVE, CR, and HTMT) in the main body of the text or as supplementary materials.

**Authors' Response:**

Thank you for your valuable comment. We appreciate your emphasis on ensuring transparency in reporting the measurement model.

In the revised manuscript, we confirm that the measurement model statistics, including standardized loadings, Average Variance Extracted (AVE), Cronbach's Alpha, Composite Reliability (CR), and Heterotrait-Monotrait Ratio (HTMT) values, are already presented in Table 4. The table is titled " Table 4. Reliability and Measurement Model Assessment" and is located in the single Supporting Material file.

The related explanatory text is provided in the revised manuscript under *Section 4.2.2. Measurement Model and Reliability Assessment Results,* on **pages 20**, highlighted in green for ease of review. The revised text in Section 4.2.2 clearly details the reliability and validity results, confirming that all constructs meet or exceed established statistical thresholds

To improve clarity and consistency, we have adjusted the abbreviations used in Table 4. Specifically, we now use EVA, CR, and HTMT in the column headings as follows:

**Observed Variable Components | Abbreviation | VIF | Standardized Loadings | EVA | Cronbach's Alpha | CR | HTMT**

Additionally, we have included a note at the end of Table 4 to define all abbreviations for ease of interpretation (see, page 21, highlighted in yellow): Note: VIF = Variance Inflation Factor; AVE = Average Variance Extracted; CR = Composite Reliability; HTMT = Heterotrait-Monotrait Ratio.

Once again, thank you for your constructive input and for helping us enhance the quality and clarity of our manuscript.

**Reviewer 2:** 2. Although the theoretical framework is robust, it lacks integration. Becker's human capital theory and North's institutional theory are both presented effectively on their own, but they are not well integrated or synthesized, which is the most important contribution of this study.

Add a synthesis paragraph outlining the potential interactions between human capital and institutions that could impact sustainability.

**Authors' Response:**

We sincerely thank Reviewer 2 for this insightful and constructive comment. In response, and in alignment with Reviewer 1’s related suggestions, we have undertaken a comprehensive revision of *Section 2 (Literature Review)*, on page 4 – 11, highlighted in blue and green.

Specifically, we have a revised subsection titled ***“2.2. Integrating Institutional and Human Capital Theories: A Dual-Lens Approach to Sustainable Transportation Development”*** (see revised manuscript, pages 5 - 11; highlighted in blue and green). This section engages both theories in dialogue, emphasizing how institutional structures are associated with the sustainable transportation development, and also integrated with capital, and vice versa.

For instance, while North’s institutional theory addresses the formal and informal rules governing behaviour, Becker’s human capital theory explains how individuals interpret and respond to these institutional contexts. We elaborate on how enabling institutions (e.g., regulatory incentives, digital infrastructure) promote the development and retention of human capital, and how skilled personnel, in turn, reinforce or challenge institutional norms through innovation, leadership, and knowledge sharing. Conversely, constraining institutions (e.g., corruption, rigid bureaucracy) can limit the impact of even highly capable individuals. In return, empowered human capital can act as a catalyst for institutional improvement.

This conceptual integration directly informed our development of a unified **research gap discussion, main contributions**, and a **consolidated set of hypotheses** (see revised manuscript, pages 9 - 11; highlighted in blue and green). The hypotheses reflect the bidirectional associations between institutional factors, human capital, and the three dimensions of sustainable transportation development, economic, social, and environmental. We have structured them to capture both direct and reciprocal relationships, as recommended.

We sincerely hope this revised and integrated approach addresses your concern and enhances the theoretical coherence and contribution of the manuscript. Thank you again for your valuable guidance.

**Reviewer 2:** 3. Definitions and operationalizations of constructs that are too general. The dichotomy of "positive" and "negative" institutional factors is overly simplistic. The subtleties of institutional economics are oversimplified by this binary classification. Think about creating multidimensional subscales (such as transparency, corruption, and enforcement) or utilizing institutional quality indices.

Items like "innovation" and "creativity," which are more closely related to firm-level capabilities than to human capital in and of themselves, are included in the human capital construct. Give a clearer explanation for their inclusion.

**Authors' Response:**

Thank you for your valuable feedback. In response to Reviewer 2’s concern regarding the binary classification of institutional factors, we have carefully and thoroughly revised the manuscript to ensure greater conceptual nuance. The revised version now clarifies that institutional factors are multidimensional and grouped into “supporting” and “challenging” categories solely for analytical purposes within the model (see page 9, highlighted in green).

We have operationalized each category using multiple validated subcomponents drawn from relevant literature and refined through expert interviews, aligning them with established institutional quality indices. Corresponding references are now provided in the first column on the right of Table 2, which is located in the single Supporting Material file. For additional clarity, a **note** has been added beneath Table 2 (see page 18 -19, highlighted in yellow): **Note:** Institutional factors are operationalized using multidimensional subscales grounded in institutional economics literature and validated through expert interviews. Each factor, initially termed INN/INP, is reframed as “Challenging” and “Supporting” to reflect functional roles. These categories encompass both formal and informal institutional elements and are not treated as a simplistic binary.

In line with this refinement, all instances of “positive” and “negative” institutional factors have been replaced with “supporting” and “challenging” (and, where contextually appropriate, “enabling” and “constraining”) throughout the manuscript. Furthermore, the hypotheses have been updated to consistently use the term “associated/association.” For example: H1: Institutional factors (supporting and challenging) are directly associated with the three dimensions of sustainable transportation development. (see pages 10 - 11, highlighted in blue).

**Reference:**

1. H. A. F. Vidotto, P. M. A. Ferenhof, R. M. Selig, and R. C. Bastos. A human capital measurement scale. J Intell Capital 2017;18(2):316-29

**In terms of human capital**

Thank you for raising this important point. We acknowledge that “innovation” and “creativity” are often associated with firm-level capabilities. However, in this study, human capital is conceptualized not merely as a set of individual attributes (e.g., education, training, leadership) but as a strategic resource that directly contributes to the development of firm-level capabilities. Specifically, we include creativity (HU6) to capture the item “Organization consistently generates new ideas,” inherited from Vidotto et al. (2017), reflecting the capacity of employees to generate novel ideas that drive innovation, an interpretation supported by prior research recognizing creativity as a key antecedent to organizational innovation.

“Based on your valuable and constructive feedback, for consistency, we have replaced the term ‘innovation’ with ‘creativity’ in the observed scale components of human capital throughout the revised manuscript.”

This conceptualization is consistent with **Dynamic Capabilities Theory** (Eisenhardt & Martin, 2000; Teece, 2007, 2020), which extends the Resource-Based View by emphasizing not only the possession of resources but also the ability to reconfigure and adapt them in response to external changes. The theory centres on three core capacities*,* sensing, seizing, and transforming*,* that underpin organizational agility and innovation. Within this framework, human capital is a critical enabler of dynamic capabilities and thus integral to firm-level performance.

Empirical studies, further support this view (see, Table 2, which is located in the single Supporting Material file), demonstrating that human capital dimensions such as education, experience, leadership, and job satisfaction play a direct role in shaping organizational capabilities and achieving sustainable outcomes, especially within the transport enterprise context.

Additionally, we have inserted a note beneath Table 2 for clarity (see page 18-19, highlighted in yellow): **Note:** Human capital (HU) is conceptualized as a multidimensional construct, including leadership, motivation, qualifications, training, satisfaction, and creativity. The creativity item reflects employees’ capacity to generate ideas and contribute to firm-level innovation, in line with prior studies that recognize creativity as a precursor to innovation.

**References:**

2. Eisenhardt KM, Martin JA. Dynamic capabilities: what are they? Strat Manag J 2000;21(10-11):1105-21.
3. Teece DJ. Hand in glove: Open innovation and the dynamic capabilities framework. Strateg Manag Rev 2020;1(2):233-53.
4. Teece DJ. Explicating dynamic capabilities: The nature and microfoundations of (sustainable) enterprise performance. Strategic Management Journal 2007;28(13):1319-50

**Reviewer 2:** 4. Ambiguity in causal inference: Despite strong statistical modelling, causality is not established. Although the authors refer to "bidirectional influence," they treat correlations as effects

Make it clear that unless there are compelling assumptions (such as experimental designs or longitudinal data), these are associations rather than causality.

**Authors' Response:**

Thank you for this valuable comment. We have taken clear steps to address the issue of causal ambiguity throughout the revised manuscript.

First, we carefully revised the terminology to ensure clarity of interpretation. Specifically, we replaced terms such as “influence” and “effect” with “association” throughout the manuscript, particularly across all hypotheses and sub-hypotheses, to better reflect the correlational nature of the findings. This change avoids implying causal relationships where they cannot be established due to the cross-sectional nature of the data. For example, as revised on pages 10 - 11 (highlighted in blue):

H1: Institutional factors (supporting and challenging) are directly associated with the three dimensions of sustainable transportation development.

H1(a1): Challenging institutional factors are negatively associated with the social dimension.

H3: Human capital is directly associated with the dimensions of sustainable transportation development.

H3(a): Human capital is positively associated with the social dimension.

Additionally, in **Section 2.3 *“****Framework and Study Mode****”*** (see page 12, highlighted in yellow), we added a dedicated note titled **“Note on Causal Inference,”** stating: This study proposes bidirectional hypotheses grounded in theory and prior empirical work; however, the use of cross-sectional data and PLS-SEM modelling does not permit definitive causal conclusions.

Second, in **Section 3 *(Methodology),*** under the subheading "Why PLS-SEM over regression or CB-SEM” (see page 18, highlighted in yellow), we noted: “However, as the study is based on cross-sectional survey data, the results reflect statistically significant associations rather than causal effects”.

Third, in **Section 4.2.3 *(Structural Model Analysis and Hypotheses Evaluation)*** (see page 22, highlighted in blue), we explicitly stated: “While these path coefficients support the hypothesized directions, they do not establish causality. Given the cross-sectional design, the results reflect significant associations rather than direct causal effects. Nonetheless, the magnitude of the standardized coefficients provides insights into the relative importance and practical implications of institutional factors and human capital in shaping sustainable transportation outcomes”.

Fourth, in **Section 5.1 *(Key Findings and Scope of Discussion)*** (see page 24, highlighted in blue), we emphasized: “It is essential to emphasize that these findings represent **statistical associations rather than causal effects,** given the study’s cross-sectional design. Nevertheless, the effect size, ranging from small to large, highlight the practical significance of both institutional and human capital factors in shaping sustainable transportation outcomes”.

Finally, in **Section 7 *(****Study Limitations and Directions for Future Research***)** (see page 32, highlighted in yellow), we reiterated: “Finally, this study employed a cross-sectional survey design, in which data were collected at a single point in time from each enterprise. While this design enables the identification of statistically significant associations between institutional factors, human capital, and transportation sustainability outcomes, it does not support causal inference. As such, the observed relationships are interpreted as associational rather than causal. Future research could enhance causal interpretation by adopting longitudinal or panel data to examine how these relationships evolve over time and respond to shifts in institutional and sustainable contexts”.

We hope that these clarifications sufficiently address your concern and clearly distinguish statistical associations from causal claims in our study.

**Reviewer 2:** 5. Nuance is required in the interpretation of results. Effect sizes and their practical significance are not discussed in the interpretation of β coefficients (such as 0.328 or - 0.154).

Include a section discussing how effect sizes - rather than just statistical significance – influence policy implications.

**Authors' Response:**

Thank you for this important observation. We have carefully revised the manuscript to incorporate more nuanced interpretations of effect sizes, with explicit reference to their **practical significance**, as well as theoretical guidance from Hair et al. (2022)/[70], who note that standardized path coefficients (β) closer to ±1 reflect stronger relationships, while those near zero are weaker. We now interpret each coefficient not only based on its statistical significance but also in terms of its practical relevance to sustainable transportation development outcomes.

Specifically:

**Section *4.2.3.(Structural Model Analysis and Hypotheses Evaluation)*, (see pages 21 - 22, highlighted in yellow)** has been revised to discuss each key β coefficient in terms of magnitude (e.g., weak, moderate, or strong) and its potential practical implications. For instance, the effect of supporting institutions on social development (β = 0.328) is now described as approaching a moderate level, with meaningful influence, while the effect on environmental development (β = 0.103) is considered weaker but still relevant. Similarly, the negative effect of challenging institutions on economic development (β = – 0.154) is interpreted as a small-to-moderate constraint, rather than merely being statistically significant. Human capital’s effect on environmental development (β = 0.609) is clearly characterized as strong and substantial.

### In **Section Discussion, 5.1 (Key Findings and Scope of Discussion), (see page 24, highlighted in blue),** we now explicitly state: “The effect size, ranging from small to large, highlight the practical significance of both institutional and human capital factors in shaping sustainable transportation outcomes”.

In **Section *5.4. (Comparison with Previous Empirical Studies),* see pages 26 - 27, highlighted in yellow,** we have carefully revised a paragraph referencing Hair et al. (2022)/[70] and used their guidelines to interpret the β coefficients. We further contextualized these magnitudes by comparing our findings with those of similar studies across multiple international settings *(see Table 5. Hypotheses Support and Structural Model Path Estimates (PLS-SEM) in the single Supporting Material file)*. For example, we emphasized that while some coefficients are small (e.g., β = 0.103), they are consistent with global findings and still practically relevant within emerging economies' complex institutional settings.

These revisions aim to respect and directly address your recommendation by embedding the interpretation of β coefficients within both a theoretical and applied framework. We hope this meets your expectations and enhances the clarity and analytical depth of the manuscript.

**Reference:**

[70] Hair JF Jr, Hult GTM, Ringle CM, Sarstedt M, Danks NP, Ray S. Partial least squares structural equation modeling (PLS-SEM) using R: A workbook. Cham: Springer; 2022. (Classroom Companion: Business). doi:10.1007/978-3-030-80519-7.

**Reviewer 2:** **Minor concerns**

**Reviewer 2:** 1. Language: The manuscript needs to be English edited because it contains awkward wording, redundant information, and excessively formal constructions. Suggest expert language polishing.

**Authors' Response:** We sincerely appreciate your comment regarding the language quality of the manuscript. In response, we undertook a comprehensive revision process. First, we carefully edited the manuscript ourselves to address awkward wording, eliminate redundant expressions, and simplify overly formal constructions. Second, the manuscript was reviewed by an English editing team to ensure improved clarity and flow. Finally, we received additional language editing support from Dr. James Bleach and Dr. Susan Keron of the Ōbex Project.

The revised version has been polished at both the sentence and paragraph levels, with close attention to consistency in terminology and overall readability. We believe the manuscript now reflects a more concise and accessible academic tone in line with the journal’s standards.

**Reviewer 2:** 2. Length: Close to 20,000 words, the paper is incredibly lengthy. Suggest condensing the literature review and discussion without sacrificing content.

**Authors' Response:**

We sincerely appreciate your comment regarding the length of the manuscript. In response, we have carefully reviewed the entire manuscript, with particular attention to the literature review and discussion sections. Redundant content has been removed, and the structure has been streamlined to enhance academic clarity and coherence, while ensuring that the substantive content is fully retained.

The revised manuscript now contains **13,208 words** in total: **10,176 words** in the main text (including author information) and **3,032 words** in the references. For comparison, the previous version had **17,612 words** in total, comprising **13,896 words** (including Tables and author information) in the main text and **3,716 words** in the references.

In the ***literature review* (see pages 4 – 12, highlighted in blue, green, yellow)** the original word count was **3,561 words**, which has now been reduced to **2,425 words**. The revisions are as follows:

- ***Section 2.1. Advancing Sustainable Transport: Key Dimensions, Innovative Approaches, and Strategic Significance,*** reduced from 716 words to 415 words.
- **Section 2.2 (Institutions) and Section 2.3 (Human Capital)** in the previous manuscript (combined length: 2,783 words) have been restructured. In the revised manuscript, they are integrated into ***Section 2.2. Integrating Institutional and Human Capital Theories: A Dual-Lens Approach to Sustainable Transportation Development*** (1,890 words).
- **Section 2.3. Framework and Study Mode** (120 words).

In the ***discussion section* (see pages 24 – 30, highlighted in yellow, blue, green)**, the original word count was **3,096 words** (including Table 6 – 9), now reduced to **1,976 words**. Additionally, the following tables have been moved to the single Supporting Material file to streamline the main text:

Table 6. Comparison of Relationship Coefficients with Previous Empirical Studies

Table 7. Challenging Institutional Factor Components Ordered by Standardized Loadings

Table 8. Supporting Institutional Factor Components Ordered by Standardized Loadings

Table 9. Human Capital Components Ordered by Standardized Loadings

The main discussion sections were revised as follows:

- Section 5.1. *Key Findings and Scope of Discussion*, reduced from 577 to 327 words
- **Section 5.2. *Unexpected Findings: Economic Development's Limited Bidirectional Association*,** reduced from 457 to 369 words
- Section 5.3. *Model Explanatory Power and Effect Sizes*, reduced from 276 to 182 words
- Section 5.4. *Comparison with Previous Empirical Studies*, reduced from 627 to 334 words (not including Table 6, Table 6 was moved to the single Supporting Material file)
- Section 5.5. *Analysis of Latent Constructs via Observed Variables,* reduced from 685 words to 397 words (not including Table 7-9, Table 7-9 were moved to the single Supporting Material file)
- **Section 5.6. *Study Contributions***, reduced from 474 to 367 words

We hope these revisions demonstrate our genuine effort to enhance the quality of the manuscript and align it with your suggestions and the journal’s standards.

**Reviewer 2:** 3. Figures and Tables: A number of the tables, such as the measurement model and survey scales, are excessively detailed and ought to be relegated to the supplemental material.

**Authors' Response:**

Thank you very much for your valuable suggestion regarding the presentation of figures and tables. In accordance with your recommendation to move excessively detailed tables, such as those on the measurement model and survey scales, to the supplemental material, we have carefully reviewed all nine tables and decided to move **all of them** to the single Supporting Material file. This decision was made to enhance the readability and conciseness of the manuscript, allowing readers to better focus on the main findings and discussions. At the same time, the manuscript text retains clear descriptions and explanations of the table contents, ensuring that readers can understand the key results and methodology without needing to refer to the tables directly.

A total of **9 tables** have been moved to the single Supporting Material file, as listed below:

S1_Table 1. Comparative Overview of Sample Sizes in Selected Empirical Studies Using PLS-SEM Across Various Fields

S2_Table 2. Overview of Observed Variables, Their Components, Validation via Literature and Interviews, and Corresponding Sources

S3_Table 3. Descriptive statistics analysis of the official survey

S4_Table 4. Reliability and Measurement Model Assessment

S5_Table 5. Hypotheses Support and Structural Model Path Estimates (PLS-SEM)

S6_Table 6. Comparison of Relationship Coefficients with Previous Empirical Studies

S7_Table 7. Challenging Institutional Factor Components Ordered by Standardized Loadings

S8_Table 8. Supporting Institutional Factor Components Ordered by Standardized Loadings

S9_Table 9. Human Capital Components Ordered by Standardized Loadings

A total of **4 figures** were already located in the single Supporting Material file in the previous version:

Figure 1. Framework for Sustainable Transportation Development

Fig 2. Framework and Study Model

Fig 3. Visual graph of descriptive statistics analysis of the official survey

Figure 4. PLS-SEM Results Visualization

In addition, the manuscript includes **three appendices**, which were also already located in the single Supporting Material file in the previous version:

S10_Appendix 1. Demographic and professional characteristics of pilot survey respondents

S11_Appendix 2. Cronbach's alpha test results of pilot test

S12_Appendix 3. Demographic and professional characteristics of offical survey respondents

We hope these revisions improve the manuscript’s organization and conciseness, and we thank you again for your helpful suggestion.

**Reviewer 2:** 4. References: Verify that all citations (such as [63], [64], and [7]) are accurate and formatted in accordance with PLOS ONE guidelines.

**Authors' Response:**

Thank you for highlighting this important point. We have carefully reviewed all in-text citations and corresponding references to ensure accuracy and consistency. In particular, the citations [63], [64], and [7] have been verified and corrected where necessary to align with the journal’s guidelines. All references have now been formatted in accordance with the PLOS ONE reference style requirements (**ICMJE (Vancouver style**). We appreciate your attention to detail and believe the revised version addresses this issue thoroughly.

**Reviewer 2: Specific comments**

**Reviewer 2:** 1. Abstract: Well-organized, although it should be made clear that "bidirectional relationships" are statistical associations rather than causative ones.

**Authors' Response:**

Thank you for this important clarification. We have revised the abstract to clearly state that the bidirectional relationships examined in this study represent statistical associations, not causative effects. This adjustment aligns the language of the abstract with the methodological approach of partial least squares structural equation modelling (PLS-SEM), which supports the analysis of associations rather than causality (see Abstract page 1, highlighted in yellow).

**Reviewer 2:** 2. Introduction: Strong context-setting in the introduction; however, to prevent reader fatigue, it could be condensed by 20% to 30%.

**Authors' Response:**

Thank you for this constructive suggestion. We have carefully reviewed and revised the Introduction section to enhance clarity and conciseness, while preserving the key context and background of the study. The original Introduction contained **1,335 words**, which has now been reduced to **934 words, a 30% reduction**. We hope this revision addresses the concern and improves the overall readability of the manuscript.

**Reviewer 2:** 3. Literature review: Needs tighter synthesis, but impressive depth. Don't use several paragraphs to reiterate what institutional theory is.

**Authors' Response:**

Thank you for this constructive suggestion. In response to your comment regarding the need for a tighter synthesis in the literature review and the avoidance of lengthy reiterations of institutional theory, we have undertaken a careful and substantive revision of the manuscript. We also considered your earlier related feedback under Minor concerns (“Length: Close to 20,000 words…”) and your observation that the theoretical framework, while robust, required stronger integration of Becker’s human capital theory and North’s institutional theory.

Accordingly, we have comprehensively revised ***Section 2 (Literature Review)*** in the revised manuscript (pages 4 - 11, highlighted in blue and green). Specifically, the previous Sections 2.2 (Institutions) and 2.3 (Human Capital) have been restructured into a new, integrated subsection: ***“2.2. Integrating Institutional and Human Capital Theories: A Dual-Lens Approach to Sustainable Transportation Development”*** (pages 5 - 11). This section directly engages both theories in dialogue, highlighting their interplay and mutual influence in shaping sustainable transportation outcomes.

For example, while North’s institutional theory addresses the formal and informal rules governing societal behaviour, Becker’s human capital theory explains how individuals interpret and respond to these institutional contexts. We elaborate on how enabling institutions (e.g., regulatory incentives, digital infrastructure) foster the growth and retention of human capital, and how skilled personnel, in turn, reinforce or challenge institutional norms through innovation, leadership, and knowledge sharing. Conversely, constraining institutions (e.g., corruption, rigid bureaucracy) can limit the effectiveness of even highly capable individuals, though empowered human capital can also catalyse institutional reform.

This conceptual integration directly informed the development of our unified research gap discussion, clarified main contributions, and consolidated set of hypotheses (pages 9 - 11). The revised hypotheses now explicitly capture the bidirectional associations between institutional factors, human capital, and the three dimensions of sustainable transportation development, economic, social, and environmental.

We believe these revisions have produced a more concise, integrated, and theoretically coherent literature review while retaining the depth you noted positively in your feedback. We sincerely thank you for your valuable guidance, which has significantly improved the clarity and contribution of this section.

**Reviewer 2:** 4. Methods: Provide a more thorough justification for sample representativeness. Is 354 of 3,908 sufficient for generalization? Explain why two stages of data collection were required as well.

**Authors' Response:**

Thank you for your valuable comment regarding the justification for sample representativeness and the rationale behind the two-stage data collection. We have carefully addressed these points in the revised manuscript.

To assess whether the sample size of 354 transportation enterprises is sufficient for generalization and robust structural modelling, we have added a new section, ***3.3 Sample Size Justification*** (see pages 14 - 15, highlighted in blue). This section applies two widely accepted methodological benchmarks from the PLS-SEM literature, as recommended by Hair et al. (2022)/[70]:

**The "10-times rule"** requires that the minimum sample size be at least ten times the maximum number of structural paths directed at a single latent construct. In our model, the maximum number of arrows pointing at any single construct is three, which implies a minimum sample size of 10 × 3 = 30. Our final sample of 354 valid responses far exceeds this threshold.

**The inverse square root method**, a more conservative and statistically rigorous guideline, suggests that for a medium effect size (path coefficient of 0.2) and a significance level of 5%, the required minimum sample size is: n_(min)_ >= (t/r)^2^ = (2.486/0/2) ^2^ = 154.5. Therefore, a minimum of 155 respondents is required. Our sample of 354 satisfies this requirement with a comfortable margin.

To further validate our approach, we conducted a comparative review of selected empirical studies employing PLS-SEM, summarized in “*Table 1. Comparative Overview of Sample Sizes in Selected Empirical Studies Using PLS-SEM Across Various Fields*” (see the single Supporting Material file). These studies, spanning sectors such as transportation SMEs, general SMEs, construction, healthcare, finance, and manufacturing, used sample sizes ranging from 64 to 350 respondents. The number of structural paths per latent construct in these models varied from 3 to 9, illustrating that sample sizes within the 150 - 350 range are widely accepted for model estimation and hypothesis testing in PLS-SEM. Together with theoretical support from Hair et al. (2022)/[70], this empirical benchmarking confirms that our sample of 354 is methodologically sufficient for both generalization and structural modelling.

Furthermore, the sample was drawn using a **stratified probability sampling** approach from a population of 3,908 transportation SMEs operating in 18 districts of Ho Chi Minh City. This represents approximately 9% of the population, satisfying external validity requirements and aligning with standards in peer-reviewed organizational research, thereby supporting the representativeness and generalizability of our findings.

Regarding the **two-stage data collection process**, a detailed explanation has been provided in ***Section 3.2. Data Collection and Bias Control*** (pages 13, highlighted in yellow). Data were collected in two distinct phases: August - October 2023 and May - June 2024, across all 18 key districts of Ho Chi Minh City. This staggered design was purposefully implemented to enhance both the robustness and validity of the dataset.

The **first phase** allowed for the early identification of key trends and any potential inconsistencies in responses. The **second phase** provided an opportunity to refine the survey administration process, address sampling imbalances, and target underrepresented respondent groups. More importantly, this staggered process also allowed the study to account for potential differences in institutional environments, human capital conditions, and operational realities, which often fluctuate in fast-changing regulatory and business contexts such as those found in Ho Chi Minh City. This design enhanced both district-level representation and the internal and external validity of findings related to sustainable transportation development.

Finally, we would like to clarify that prior to the main data collection, a **pilot study** was conducted with 50 transportation enterprise managers across all 18 districts. The pilot aimed to ensure the reliability, clarity, and contextual appropriateness of the questionnaire items, and directly informed the revisions made for the final survey instrument.

**Reviewer 2:** 5. Results: Tables are understandable, but there is no explanation of effect size.

**Authors' Response:**

Thank you for your valuable comment. In response, we have added a detailed explanation of the effect size (f²) in the revised manuscript to enhance the interpretation of the structural model results. Specifically, the new subsection ***“Effect Size (f²) Evaluation”*** has been added under *4.2.3. Structural Model Analysis and Hypotheses Evaluation* (see **pages 22 - 23**, highlighted in green). This section describes the method of f² calculation following Hair et al. (2022)/[70], reports the R² values from the exclusion models (INN, INP, and HU), and interprets the resulting nine f² values.

Additionally, effect size values (f²) have been incorporated into **Table 5**, now provided in the single Supporting Material file ***(S5_*** ***Table 5. Hypotheses Support and Structural Model Path Estimates (PLS-SEM)).*** Columns 2 and 3 from the right show the effect size interpretation and f² values for each path, clearly indicating which relationships are of large, moderate, small, or negligible effect.

We hope these revisions sufficiently address your comment and improve the clarity and quality of the results section.

**Reviewer 2:** 6. Discussion: The introduction and this section are similar. Highlight the most important discoveries and make a clear connection between them and your research questions and hypotheses.

**Authors' Response:**

Thank you for your valuable comment. We have carefully revised the structure and content of the Discussion section to avoid overlap with the Introduction and to more clearly highlight the key findings. The revised Discussion explicitly links these findings to the research questions and hypotheses, thereby improving clarity and coherence.

In particular, we have removed the former subsection 5.4 *“Scope of Discussion*”, which previously overlapped with the Introduction. In its place, we have substantially revised subsection **5.1**, now titled ***“5.1. Key Findings and Scope of Discussion”*** (see **pages 23 - 24**, highlighted in yellow, green, and blue). This revised section presents a focused synthesis of the main findings, emphasizing their theoretical and empirical relevance.

Specifically, we clearly outline how the results support or do not support each hypothesis (e.g., H1–H4 and sub-hypotheses), and how these relate to the study’s core research questions. We have also clarified the bidirectional **associational** nature of the relationships, especially between institutional factors, human capital, and sustainability dimensions in transportation enterprises.

Moreover, subsection ***5.2 “Unexpected Findings: Economic Development's Limited Bidirectional Association”*** (see **pages 25 - 26**, highlighted in yellow), to draw attention to one of the study’s most significant and novel discoveries. This section provides contextual explanations for the absence of expected associations and offers theoretical contributions that distinguish our findings from existing literature.

Finally, we have clarified throughout that the reported relationships are **statistical associations** rather than causal effects, due to the study’s cross-sectional design. These revisions ensure that the Discussion section now provides a critical, hypothesis-driven interpretation of the findings, distinct from the Introduction, and better aligned with the study’s conceptual framework.

Thank you once again for your time, thoughtful engagement, and support throughout the review process. We hope the revised manuscript meets your expectations and look forward to your feedback.

Warm regards,
**Vu Thi Kim Hanh**

**Nguyen Hong Nga**
