# Supplementary material for: Investigating sustainable development in transportation enterprises: Novel insights from new institutional economics and human capital theory. Evidence from HCM, Vietnam
Source: PLoS One. 2025 Nov 17;20(11):e0333393. doi: 10.1371/journal.pone.0333393 (PMC12622828; doi:10.1371/journal.pone.0333393)
Supplement: Attachment — Submitted filename: Response to reviewer comments by authors (PONE-D-25-32321).pdf [file pone.0333393.s009.pdf]

Major Concerns

1. **Use of PLS-SEM and Model Justification**

   - **My comment:** Clarify whether study is exploratory or confirmatory; justify PLS-SEM use.
   - **Authors' response:** Clearly state an exploratory-confirmatory hybrid approach justified through theory-building and hypothesis testing; referenced Hair et al. (2022).
   - **Validation:** Thorough and justified explanation reflecting current best practice in PLS-SEM methodology; adequately clarifies original ambiguity.

2. **Measurement Model Transparency**

   - **My comment:** Include measurement model tables (loadings, AVE, CR, HTMT) in main or supplemental materials.
   - **Authors' response:** Provided comprehensive measurement statistics in Table 4 of Supporting Material, with detailed explanatory text in the manuscript; improved abbreviations and definitions for clarity.
   - **Validation:** Fully addresses transparency requirements; improved clarity and accessibility of key measurement statistics.

3. **Integration of Becker's Human Capital and North's Institutional Theory**

   - **My comment:** Theoretical integration lacking; add synthesis on human capital and institutional interaction impacting sustainability.
   - **Authors' response:** Developed a new dedicated integrated subsection in literature review linking the two theories bidirectionally with examples; informed research gaps and hypotheses.
   - **Validation:** Major improvement yielding coherent, integrated theoretical framework; directly responds to the core concern.

4. **Construct Definitions and Operationalizations**

   - **My comment:** Binary classification of institutional factors too simplistic; clarify innovation and creativity in human capital.
   - **Authors' response:** Reframed institutional factors as multidimensional "supporting" and "challenging" with nuanced subscales validated by literature and interviews. Clarified creativity as a strategic resource tied to innovation per Dynamic Capabilities Theory; replaced "innovation" wording with "creativity."
   - **Validation:** Substantial conceptual refinement and justification aligned with literature improve construct validity and operationalization rigor.

5. **Causal Inference Ambiguity**

   - **My comment:** Clarify associations not causal effects due to study design.
   - **Authors' response:** Systematically replaced "causal" terms with "association." Included explicit disclaimers on cross-sectional design and non-causal interpretations throughout manuscript.
   - **Validation:** Addresses fundamental methodological concern; language adjustments clear and consistent.

6. **Effect Size Interpretation**

   - **My comment:** Interpret effect sizes (β, f^2) in terms of practical and policy significance, not just statistical significance.
   - **Authors' response:** Added detailed effect size discussion, interpreting magnitude of coefficients in results and discussion sections, with references to Hair et al. (2022) standards. Linked findings to practical implications.
   - **Validation:** Satisfies need for nuanced interpretation; improves relevance and reader understanding.

Minor Concerns

1. **Language Quality**

   - **Authors' response:** Undertook comprehensive editing including professional language polishing by experts.
   - **Validation:** Likely improved readability and style as recommended.

2. **Manuscript Length**

   - **Authors' response:** Reduced overall manuscript length by >25%, condensed literature and discussion, moved extensive tables to supplements.
   - **Validation:** Enhances conciseness while retaining content; aligns with journal standards.`

3. **Tables and Figures**

   - **Authors' response:** Moved all detailed tables and some figures to supplemental files, retained clear narrative in main text.
   - **Validation:** Improves readability; consistent with best practices.

4. **Reference Accuracy**

   - **Authors' response:** Reviewed, corrected, and formatted all citations per journal guidelines.
   - **Validation:** Thorough citation management responds well to the suggestion.

5. **Abstract and Introduction**

   - **Authors' response:** Clarified bidirectional relationships as statistical associations in abstract; reduced introduction by ~30%.
   - **Validation:** Meets clarity and conciseness goals, improving reader engagement.

6. **Literature Review Synthesis**

   - **Authors' response:** Tighter synthesis of institutional theory with human capital theory; avoided repetition and redundancy.
   - **Validation:** Demonstrable enhancement of theoretical coherence and depth.

7. **Sample Representativeness and Data Collection**

- **Authors' response:** Added dedicated section justifying sample size by established PLS-SEM criteria, explained two-stage data collection to improve robustness and representativeness; detailed nonresponse and method bias controls.
- **Validation:** Strong justification improving reader confidence in study reliability and validity.

8. **Results Presentation**

- **Authors' response:** Added effect size explanation (f^2) with interpretations and incorporated in results table.
- **Validation:** Strengthens interpretation and practical significance of findings.

9. **Discussion Clarity**

- **Authors' response:** Reorganized discussion to avoid overlap with introduction; highlighted key findings explicitly linked to hypotheses; addressed unexpected findings.
- **Validation:** Enhances focus and theoretical alignment; improves manuscript coherence.
